# Fluctuations of Hi-Hat Timing and Dynamics in a Virtuoso Drum Track of a Popular Music Recording

**DOI:** 10.1371/journal.pone.0127902

**Published:** 2015-06-03

**Authors:** Esa Räsänen, Otto Pulkkinen, Tuomas Virtanen, Manfred Zollner, Holger Hennig

**Affiliations:** 1 Department of Physics, Tampere University of Technology, FI-33101 Tampere, Finland; 2 Department of Physics, Harvard University, Cambridge, Massachusetts 02138, USA; 3 Department of Signal Processing, Tampere University of Technology, FI-33101 Tampere, Finland; 4 Electro-Acoustic Laboratory, Regensburg University of Applied Sciences, D-93025 Regensburg, Germany; 5 Max Planck Institute for Dynamics and Self-Organization (MPI DS) Göttingen, Am Fassberg 17, D-37077 Göttingen, Germany; University of California, Merced, UNITED STATES

## Abstract

Long-range correlated temporal fluctuations in the beats of musical rhythms are an inevitable consequence of human action. According to recent studies, such fluctuations also lead to a favored listening experience. The scaling laws of amplitude variations in rhythms, however, are widely unknown. Here we use highly sensitive onset detection and time series analysis to study the amplitude and temporal fluctuations of Jeff Porcaro’s one-handed hi-hat pattern in “I Keep Forgettin’”—one of the most renowned 16th note patterns in modern drumming. We show that fluctuations of hi-hat amplitudes and interbeat intervals (times between hits) have clear long-range correlations and short-range anticorrelations separated by a characteristic time scale. In addition, we detect subtle features in Porcaro’s drumming such as small drifts in the 16th note pulse and non-trivial periodic two-bar patterns in both hi-hat amplitudes and intervals. Through this investigation we introduce a step towards statistical studies of the 20th and 21st century music recordings in the framework of complex systems. Our analysis has direct applications to the development of drum machines and to drumming pedagogy.

## Introduction

Astonishingly many dynamical or complex systems in various branches of physics, biology, and economics show 1/*f* fluctuations [1] often called as *fractal*. 1/*f*-type noise is present even in the most obvious human-generated time-series such as heart beat [2–4], gait [5], and tapping or drumming [6–10].

In an early study, Voss [11] found that the musical pitch and loudness follow 1/*f* fluctuations. Loudness fluctuations were studied by analyzing, e.g., a recording of Bach’s 1st Brandenburg Concerto. In this case, however, the fluctuations were taken from the full audio signal in a ‘continuous’ sense. Later on, fractal analysis of loudness variations has been used to classify genres and styles of music [12–14].

Fractal analysis on human musical *rhythms* has been done only very recently [6, 7]. Remarkably, clear long-range correlated (LRC) fluctuations were consistently found in various rhythmic tasks, albeit often outside the 1/*f* regime (see below for the mathematical definitions). Another important finding in the subsequent perception study was the fact that the listeners had a statistically significant preference for ‘1/*f* humanized’ samples over ‘white-noise humanized’ samples. Furthermore, it was shown very recently that rhythms between individuals are subject to scale-free cross correlations [15]. These findings underline the subtlety of a rhythmic interplay in musical performances and in their perception.

Despite the advances in the quantification of human rhythms, the statistical (fractal) properties of rhythmic fluctuations in the 20th and 21st century music recordings have not been analyzed in detail. It should be noted that in previous studies on tapping and drumming [1, 6, 8, 9, 15] the experiments were either (i) conducted in a ‘clean’ laboratory environment or (ii) individual drumming tracks were used where a metronome was present. A metronome leads to a constant pace and defines a constant grid for audio engineers. However, it qualitatively changes the behavior on both short and long time scales [1, 10, 15]. In other studies, classical piano music (without drums or metronome) was subject to fractal analysis, where clear signatures of 1/*f* tempo fluctuations were found [16, 17].

Here we take the first step to fill the gap between carefully designed experimental studies under controlled conditions [8] and recorded drumming under real-world conditions. In the latter case, further studies are needed to determine and classify fluctuations in both interbeat intervals and beat amplitudes. To the best of our knowledge, the correlation properties of amplitude (i.e., loudness) fluctuations of beats in rhythms have not been scrutinized as yet. Moreover, it is worth studying whether “human” fluctuations contribute to the *groove*—sometimes defined as the subjective experience of wanting to move rhythmically when listening to music [8, 18–24]. Previously, it has been found that microtiming deviations without LRCs do not affect the listener groove ratings [19, 20], or even correlate negatively with them [23, 24]. On the other hand, groove ratings can be changed with other aspects in the rhythmic structure, e.g., with syncopation [21, 22]. Here we focus on timing and loudness variations that occur naturally when a drummer plays to a piece of music, and suggest that they may also contribute to the groove. However, we do not provide an exhaustive treatment of groove from a musicological point of view.

In this work, we thoroughly analyze a one-handed hi-hat drumming pattern of a musical masterpiece recorded in 1982 [25]. We use sensitive signal-analysis tools to detect the onset times of hi-hat hits of the song with a millisecond accuracy. The onset of a hit is defined as the time when the hit begins. Once the onsets are detected, we carry out time-series analysis on the sequence of onset times. Firstly, we examine the drift of the 16th note pulse that strongly correlates with the parts of the song and shows that the drum track was recorded without a metronome. Secondly, the fluctuations of the 16th note hi-hat intervals as well as hit amplitudes are subjected to detrended fluctuation analysis (DFA) and power-spectrum study, which clearly show the existence of LRCs in both cases. However, a Poincaré plot of the interval variability [*i*:th interval versus (*i*+1):th interval] shows strong lag-1 anticorrelations. This suggests motor delays in the 16th note hi-hat pulse in accordance with previous behavioral data and models. Finally, we demonstrate that each repetitive phrase of the song, consisting of two bars and 32 hi-hat hits, has a specific amplitude pattern. Also the hit intervals show positive correlations across phrases. Interestingly, the phrase—as defined above—seems to correspond to the time scale that separates the LRCs (at longer times) and anticorrelations (at shorter times). The paper is concluded with a discussion on implications and possible follow-ups of the present study.

## Materials and Methods

### Object of study

In our analysis, we focus on one song, *I Keep Forgettin’* by Michael McDonald recorded in 1982 [25]. It is a low-mid tempo (96 quarter-note beats per minute) pop-soul song with a well-known 16th note hi-hat drum pattern played by Jeff Porcaro [26]. Jeff Porcaro (1954–1992) was one of the most renowned drummers of his time; a session musician behind recordings of, e.g., Michael Jackson and Madonna, and a member of major rock bands Steely Dan and TOTO. One of Porcaro’s trademarks was his single-handed hi-hat technique that he used to play 16th note patterns with a particularly smooth and groovy feel [27]. *I Keep Forgettin’* features this technique in its most recognizable form. In his instructional drumming video Porcaro comments on his hi-hat playing in this song [27]:

*“I like the single-handed method, because it’s a lot smoother feel. For instance in the Michael McDonald record ‘I Keep Forgettin”, I tried doing the alternating stroke method of doing 16ths, and it sounded just too stiff and staccato for me.”*

The comment makes an intriguing starting point for the present study from a musicological point of view. The results below reflect Porcaro’s comment in the sense that there is a smooth and subtle modulation in his single-handed hi-hat playing. It is commonly agreed by drummers that, e.g., modulations in hi-hat accents are important in the generation of the “groove”, and Jeff Porcaro is highly respected for this ability. In addition, we find LRCs in both interval and amplitude variations. To find out whether LRCs exist also in *two-handed* patterns is, however, a subject of future studies.

**Fig 1:**
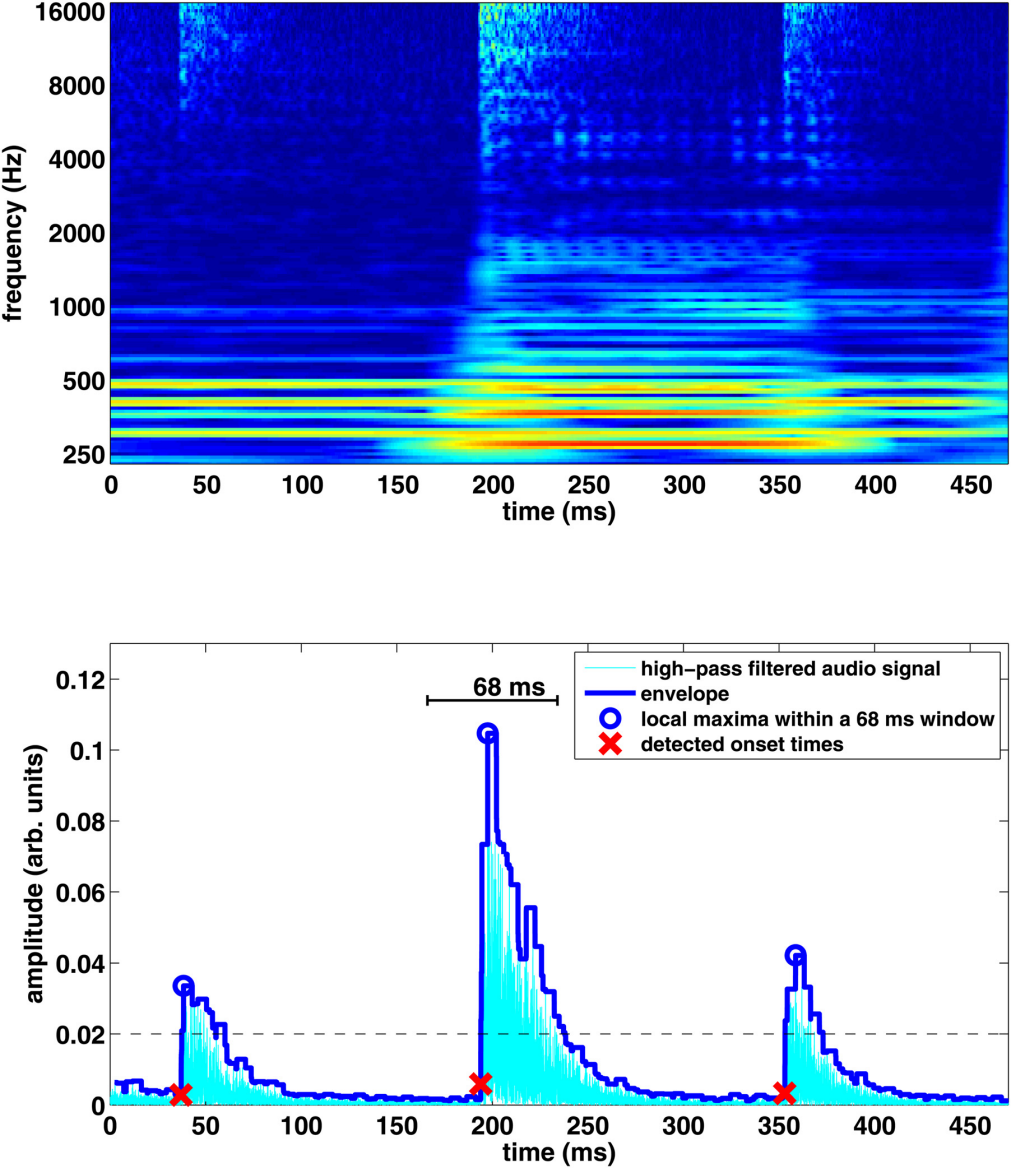
Upper panel: Audio signal of a short clip of the song presented as a spectrogram. The bright branches at high frequencies correspond to the hi-hat beats. Lower panel: Cross section of the spectrogram with the envelope, amplitude threshold (dashed line), and the detected onset times (crosses).

From a physical and mathematical point of view, the selected song is well suited for quantitative analysis for the following reasons. First, the large number of onsets in hi-hats played on the 16th notes allows sufficiently reliable fractal analysis with DFA. Secondly, the song is strongly driven by drums and bass that dominate the instrumentation in most parts of the recording. This helps the precise determination of the onset times. In general, hi-hats suit well for onset analysis due to their high frequency range as shown below (Fig 1).

### Onset analysis

In the original recording, all instruments are mixed together. To select a specific component from the complete song, here the hi-hat hits, we use frequency filters and semi-automated sensitive onset detection. The onset times of hi-hats are obtained by first applying a computational onset detection algorithm on the audio signal, and then manually editing the onset positions. The original audio signal is an uncompressed stereo WAV file extracted from the original compact disc [25] having sampling frequency of 44.1 kHz and 16 bits per sample. There exist established algorithms for onset detection of musical sounds [28]. In this study we are interested in the onsets of hi-hats only, and therefore generic onset estimation algorithms are not applicable for our purpose. Instead, we implemented an onset detection algorithm for hi-hats in MATLAB [29].

The main challenge in the onset analysis is the polytimbral nature of the material: the signal is a mixdown of multiple instruments that overlap with each other in time and frequency. Hi-hats have most of their energy at high frequencies, whereas most of the other instruments are dominated by low frequencies. In order to make the hi-hat sounds more prominent for the subsequent onset estimation, the signal was first filtered with a 100th-order FIR filter with a cutoff frequency of 8 kHz. The delay caused by the filter was compensated by shifting the signal.

Onsets are most clearly visible in the amplitude envelope of the signal as shown in Fig 1. In the automatic onset analysis, the envelope of the filtered signal is calculated by finding the maximum of the absolute value of the signal within a 200-sample (4.5 ms) window centered at each sample. Hi-hat instances are found as the local maxima of the envelope, higher than a threshold that was manually tuned not to discard any real hi-hat instances.

The onset time of each hi-hat instance is found by examining a 1500-sample (34 ms) window before each hi-hat instance time, assuming that the hi-hat sound starts at most 34 ms before its maximum amplitude. The onset time is defined to be the time when the amplitude of the envelope rises above 10% of the maximum amplitude of the instance. This *percent method* has successfully been used to extract onset times of other types of instruments as well [30]. The above method works well for estimating onsets when no other instruments were present. An example of successful onset analysis based on the approach described above is illustrated in Fig 1. However, interference from other instruments may rise the general level of the envelope above 10% of the maximum. In this case, the threshold was doubled until a rise from below to above the envelope was found.

Sounds produced by other instruments can produce erroneous onset estimates. Here we use an automatic constraint that the interval between true hi-hat onsets can deviate at most ±20 ms from 157 ms, which is the average interval of the 16th notes. However, there are cases when instruments (mostly snare drums and cymbals) occur simultaneously with a hi-hat sound in the same frequency range and make the determination of the exact onset time impossible. In those cases the onsets were omitted from further analysis.

Finally, the onset candidates were manually examined to confirm their correctness. First, the highpass-filtered signal and estimated onset times were visually examined in an audio editor while listening to the original and filtered signals. Second, a “click” track was produced by generating a synthesized click sound at the estimated onset times. The original and the click track were listened alternately to spot any instances where the perceived onset times differed from each other. As a result of the examination, the onset times were manually changed to match with the perceived hi-hat onset times. The above methods were used in small segments of the signal at a time, and each segment was examined multiple times to verify the correctness of the onset times.

In total we detected 931 hi-hat onsets (see S1 Dataset). *All* the onsets were used for the analysis of *amplitudes* below. For the analysis of hi-hat interbeat intervals, however, we included only the clearly detected 16th note intervals. Therefore, we needed to omit the intervals having a missing onset (or many of them) in between. Also the 8th note intervals with an open hi-hat, often played at the end of the phrase consisting of two bars (see below), were omitted. The total number of detected 16th note hi-hat intervals is thus 708, leading to a considerably large detection rate of 76% in the intervals with respect to the total number of onsets.

### Detrended fluctuation analysis

DFA is a widely used method in time-series analysis to study long-range correlations, particularly the 1/*f* noise [31]. Several studies over the past 20 years have shown the usefulness of DFA to determine fractal properties of non-stationary time series [6, 13, 31–33]. Outside the time domain, it has been used to study, e.g., DNA structures [34], and very recently also magnetoconductance of chaotic quantum dots by some of the present authors [35]. The reliability of DFA against alternative methods to determine fractal properties has been quantitatively confirmed by Pilgram and Kaplan [36].

The 1/*f* noise essentially means that the power spectrum of a signal *f*(*i*) is of a power-law form *S*(*f*) ∼ 1/*f* ^*β*^ with *β* ∼ 1. This is often referred to as pink or flicker noise that has intermediate predictability between (i) white noise with *β* = 0 and no correlation between consecutive values and (ii) Brownian motion with *β* = 2 and strongly correlated values generated by uncorrelated consecutive *increments*[1]. In this context, 1/*f* fluctuations are often called *fractal*, for *β* corresponds to the self-similarity parameter (Hurst exponent) *α*, which describes the temporal scaling of a signal *X*(*t*) in a statistical sense: *X*(*bt*) = *b* ^*α*^ *X*(*t*), where *b* is a scaling factor. In turn, *α* corresponds also to the exponent in DFA (see below). In the DFA context, *α* and *β* are related as *β* = 2*α*−1 [37], and fluctuations leading to 0.5 < *α* ≤ 1.5 are generally referred to as *long-range correlated* (LRC). Anticorrelations are present for −0.5 < *α* < 0.5. Generally, we speak of the 1/*f* regime when *α* = *β* = 1 within statistical errors.

We apply DFA to (i) the fluctuations of the interbeat intervals (from the mean) and (ii) the fluctuations of the onset amplitudes. In the following we exemplify the conventional DFA procedure [31, 32] for the former case. In the notation we partly follow Ref. [38], where DFA was applied to rainfall and streamflow data. The onset times are denoted by *f*(*i*), so that the set of interbeat intervals becomes *τ*(*i*) = *f*(*i*+1)−*f*(*i*). Next, we subtract the mean of the intervals ⟨*τ*⟩ to obtain a set of the fluctuations of the intervals from the mean, i.e., Δ*τ*(*i*) = *τ*(*i*)−⟨*τ*⟩. Our interest lies now in the (possible) LRCs in Δ*τ*(*i*). To this end, we first integrate the series by calculating a function

$$\begin{array}{c} \Delta y\left(i\right)=\sum_{j=1}^{i}\Delta \tau \left(j\right)\;\text{for}\;i=1,2,\ldots ,N, \end{array}$$ (1)

where *N* is the number of data points. Next, we divide the *i* axis into *N*/*s* non-overlapping windows each consisting of *s* data points. In each window, a least-squares line *y*_*s*_(*i*)—that represents the trend in the window – is fit to *y*(*i*) and the residuals *y*(*i*)−*y*_*s*_(*i*) are calculated (detrending). Thus, we use *linear* detrending; quadratic (or higher order) detrending did not lead to a qualitative difference. The root-mean-square fluctuations for a window of size *s* are calculated by

$$\begin{array}{c} F_{k}\left(s\right)=\sqrt{\frac{1}{s}\sum_{i=ks+1}^{ks+s}{\left[y\left(i\right)-y_{s}\left(i\right)\right]}^{2}}\;\text{for}\;k=0,1,\ldots ,\frac{N}{s}-1. \end{array}$$ (2)

Finally, we take a mean value over all *N*/*s* elements of *F*_*k*_(*s*) to obtain *F*(*s*) = ⟨*F*_*k*_(*s*)⟩. The procedure thus yields a relationship between the average fluctuation within a certain window size and the window size itself.

We can now examine whether *F*(*s*) scales as *F*(*s*) ∝ *s* ^*α*^, where the scaling (DFA) exponent *α* is the slope of the line relating log*F*(*s*) to log*s*. The white noise and the Brown noise (integrated white noise) correspond to *α* = 0.5 and *α* = 1.5, respectively, whereas 0.5 < *α* ≤ 1.5 indicates LRC, and the special case of flicker noise *α* = 1 corresponds to 1/*f* behavior.

In this work, DFA results are supplemented by the globally detrended power spectral density (gPSD) analysis described in detail in Ref. [15]. It is a modification of the conventional PSD method and includes prior detrending with higher-order polynomials (beyond linear global detrending). Higher-order polynomial detrending has proven to be crucial when nonstationary time series—here, recordings without the metronome—are analyzed. This is expected to be even more important when real-world recordings are studied as in the present work.

We point out that DFA as well as gPSD are subject to intrinsic errors analyzed in detail by Pilgram and Kaplan [36]. These errors in the estimate of the scaling exponent do not include the numerical error of the least-square fitting procedure. In practice we expect the errors for our data sets to be below ∼ 10%, which does not lead to a qualitative difference in the interpretation of the results.

## Results

### Statistical properties and the drift

**Fig 2:**
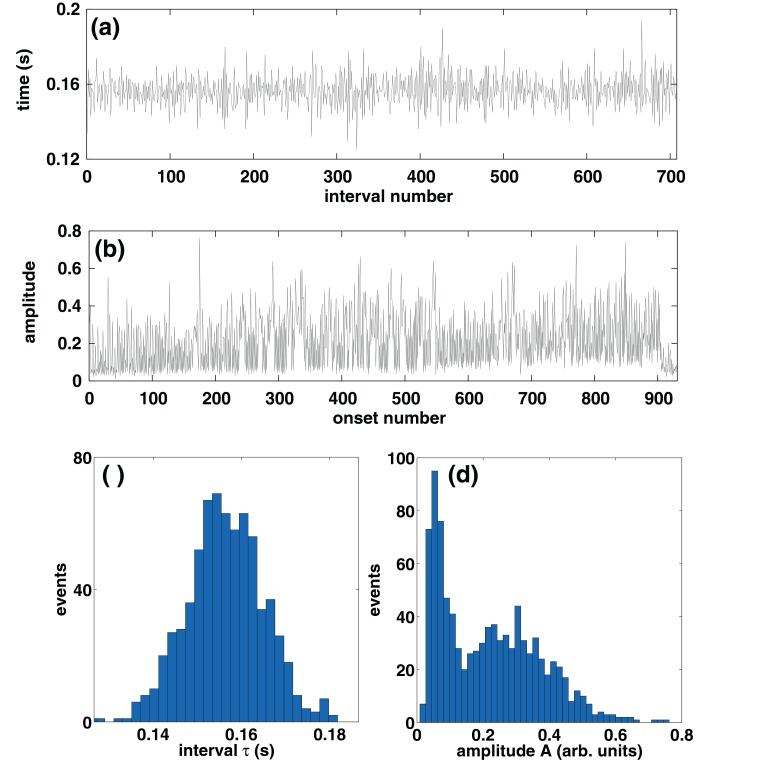
(a) Detected 16th note intervals in the song. (b) Detected onset amplitudes. (c-d) Corresponding statistical distributions of (a) and (b), respectively. The distribution of the intervals resemble a Gaussian distribution with a mean value 157 ms and a standard deviation of 8.7 ms.

The extracted hi-hat interbeat intervals (in seconds) and the amplitudes (in arbitrary units) of all the detected onsets are shown in Fig 2a and 2b, respectively. As we include only the 16th note intervals (see the onset analysis above), there are fewer data points in (a) than in (b). The intervals deviate around the mean value ⟨*τ*⟩ ≈ 156.6 ms. The qualitative shapes of the curves, especially the intervals in Fig 2a, already suggest possible LRCs, but this issue is analyzed in detail in the next section.

The statistical distributions are shown in Fig 2c and 2d, respectively. As expected, the intervals follow a Gaussian-type distribution with a standard deviation of 8.7 ms from the mean value. A chi-squared test confirms the Gaussian shape, and a skewness test indicates strong left-right symmetry with a minor, non-significant positive skewness. For comparison, in Ref. [6] the drummer had a larger standard deviation 15.6 ms when trying to synchronize with the metronome at 180 beats per minute. In both cases, however, the distributions have a Gaussian form. On the other hand, the hi-hat hit amplitudes shown in Fig 2d seem to consist of two overlapping distributions. This results from intentional accents on the every second 16th note as demonstrated below in more detail.

**Fig 3:**
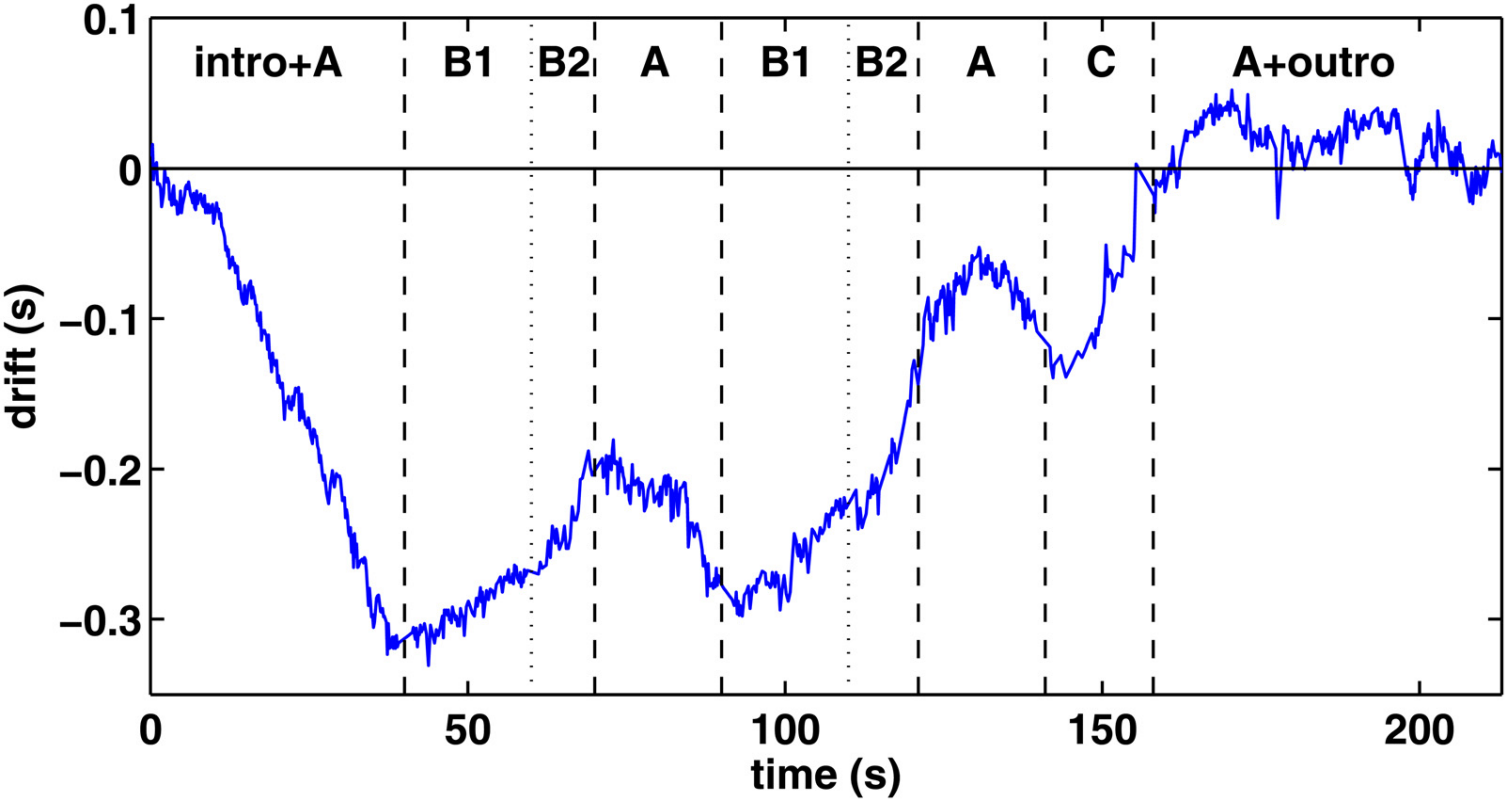
Drift of the sixteenth note pulse, i.e., the deviation from an imaginary metronome during the song. A, B1, B2, and C mark different parts of the song (see text).

In Fig 3 we show the drift of the 16th note pulse during the song. The drift *d*(*i*) can be found by comparing the time *t*(*i*) = *i*⟨*τ*⟩ to the cumulative sum of the intervals at that time, i.e,

$$\begin{array}{c} d\left(i\right)=\sum_{j=1}^{i}\tau \left(j\right)-i\left\langle \tau \right\rangle . \end{array}$$ (3)

It should be noted that the plot has been adjusted according to the missing onsets that lead to larger intervals in the data. As these larger intervals are practically close to multiples of the 16th note intervals, we have included them in the drift plot by dividing them by the closest multiple of the average 16th note interval. However, these parts can generate irregular steps in the data. They are clearly visible close to the end of the song, where there are strong orchestral parts that hinder the detection of onsets.

From the drift in Fig 3 we can make two interesting observations. First, it seems obvious that Porcaro recorded the track *without* a metronome, i.e., a click track commonly used by drummers. This can be deduced from a drift in a 300 ms scale over very long periods. Although drummers often “wander” around the click track intentionally, our example does not resemble such behavior. This finding is in line what has been commented by Grammy Award winning musician, producer, and recording engineer Jay Graydon [39], who has recorded with Porcaro: *“When playing with Jeff, better not to use a click since he played inside the cracks and his time float is what made him great.”*[40] According to Porcaro himself, the recorded track was “take two” [41].

Secondly, in Fig 3 we have marked the parts of the song (intro, A, B1, B2, C, outro) and separated them by vertical lines. Most lines coincide with changes in the slope of the drift. For example, the first vertical line at 40*s* corresponds to the beginning of the B1-part, “Every time you’re near…” [25], where the 16th note pulse (and thus the tempo of the song) starts to accelerate slightly. Further acceleration occurs at 60*s* until the A part starts ten seconds later – again with a negative slope of the drift as in the first A part in the beginning of the song. However, it is important to note that these changes in the pulse are practically non-audible to the ear.

### Fractal analysis

**Fig 4:**
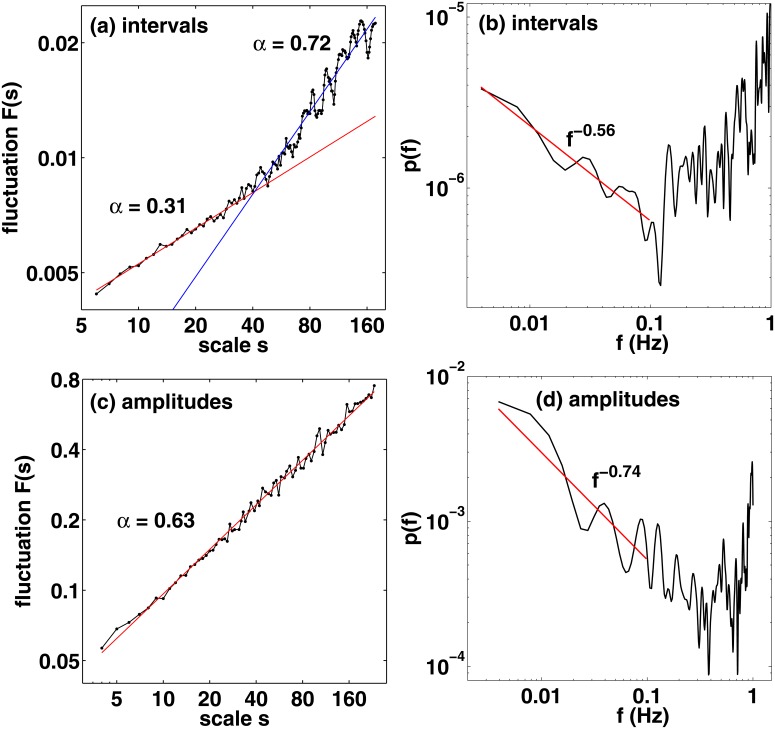
Results from the DFA (left panel) and power spectral analysis (right panel) for the fluctuations of the interbeat intervals (a, b) and the beat amplitudes (c, d).

Next we turn our attention to the DFA and power spectral analysis, starting from the fluctuations of the 16th note intervals in Fig 4a and 4b. The fluctuations *F*(*s*) show a relatively sharp kink at *s* ≈ 30…50, corresponding to a time scale *s*⟨*τ*⟩ ∼ 5…8 seconds. On a smaller scale we find a DFA exponent of *α* = 0.31 indicating anticorrelations. This is in line with previous studies on finger tapping and playing simple rhythms [1, 8, 9, 15] (see also below). In contrast, on a larger time scale the existence of LRC fluctuations can be confirmed with *α* = 0.72. This is one of the main findings in this work that confirms the existence of rhythmic LRC fluctuations outside laboratory conditions and/or without a metronome, in particular, in a recorded piece of music. The data points in Fig 4a fit well on straight lines, and the fitting is relatively stable against both the order (1st, 2nd) of DFA and the number of windows. The results in the power spectrum in Fig 4b, where we used linear detrending and a window size of 1/4 of the data, support the conclusions of DFA.

For the amplitudes, DFA yields a single exponent of *α* = 0.63 with a very good fit as shown in Fig 4b. Thus, as our second important finding, the amplitudes—or dynamics in drumming terminology—show clear LRC fluctuations, albeit they are not particularly strong (i.e., not very close to the 1/*f* limit with *α* = 1). As shown in the power spectrum in Fig 4d, generated with linear detrending and a window size of 1/4 of the data, shows a change of slope at very high frequencies. Amplitude patterns in this regime are analyzed in detail in the next section.

**Fig 5:**
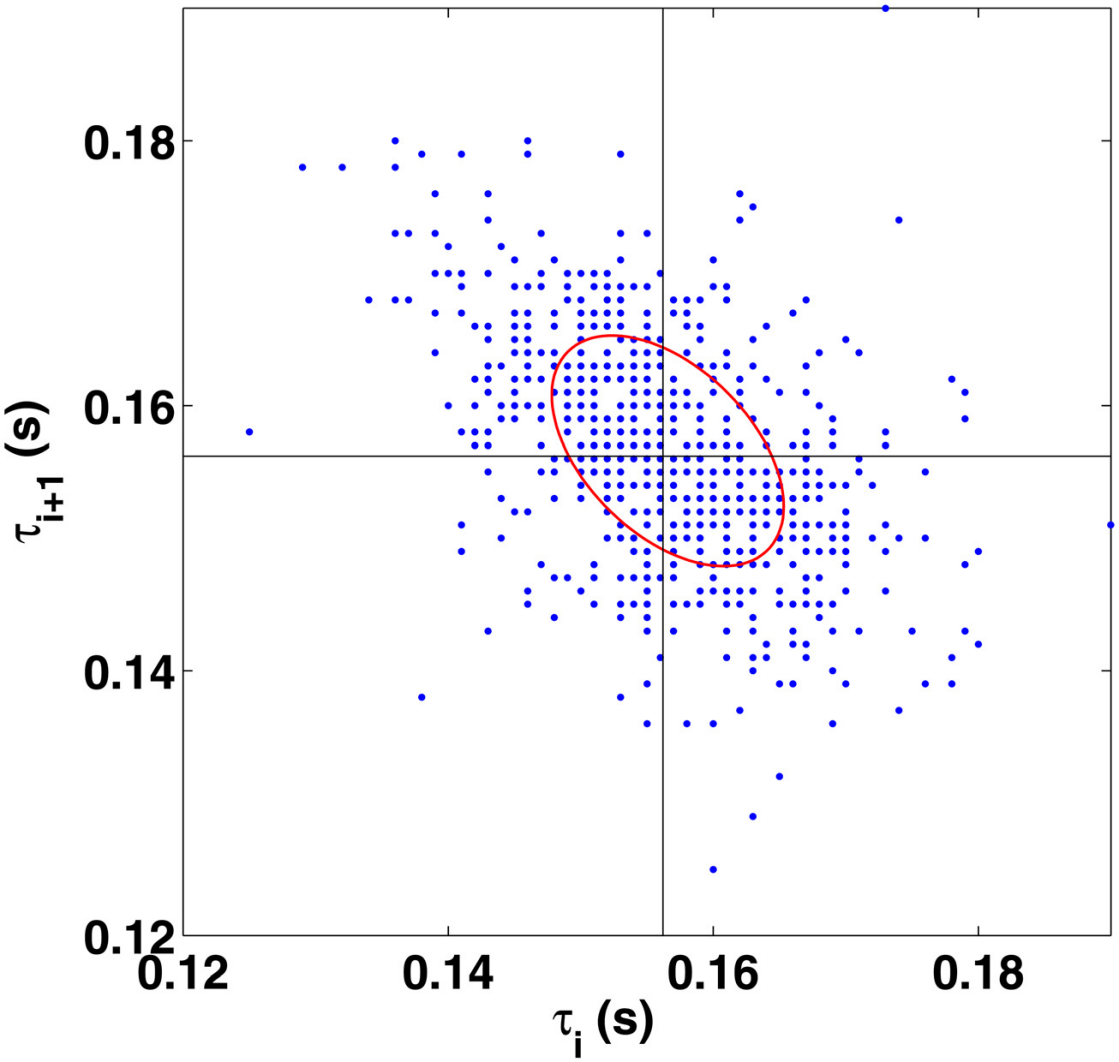
Poincaré plot (return map) of the interbeat intervals that map the i:th interval to the following one. The data shows negative correlations with Pearson coefficient −0.48.

Turning back to the interval analysis, it is interesting to note that the time scale of the changing slope in Fig 4a roughly corresponds to the two-bar phrase of the song consisting of 32 hi-hat notes and the characteristic bassline of the song. In the power spectrum, the slope changes at ∼ 0.1 Hz, which is the same order of magnitude found for finger tapping [1] and simple rhythms [15]. At higher frequencies the spectrum whitens and might even become anticorrelated. In particular, we find clear lag-1 anticorrelations as demonstrated by the Poincaré map [42] in Fig 5. The map shows consecutive interbeat intervals, *τ*_*i*_ versus *τ*_*i*+1_, sometimes referred to as return times. The distribution of the points indicates a negative correlation, which can be confirmed from the calculated Pearson correlation coefficient that has a value −0.48. The semiaxes in the fitted ellipse correspond to the double of the standard deviations.

The lag-1 anticorrelations essentially mean that, statistically, long intervals tend to be followed by short intervals and vice versa in order to maintain a given tempo. This effect has been found before, and it has been included in the models of Wing and Kristofferson [43] for a 1/*f* inner clock and Gilden *et al.* for a white-noise inner clock. A detailed analysis and discussion of the source of white and pink noise in human behavioral data can be found, e.g., in Ref. [44]. Lag-1 anticorrelations have recently been found also when two musicians interact [15]. However, lag-1 anticorrelations decay exponentially over time, and hence they are seen only for high frequencies in the power spectral density.

Interestingly, the Poincaré map in Fig 5 is very different from the corresponding plot for consecutive heartbeat intervals, which show a positive correlation in the Poincaré map [42] in the case of healthy subjects. Such a correlation has been recently found even for stem cell-derived cardiomyocytes [45]. The positive correlation here simply means that if a certain hit is behind the mean occurrence, it is (statistically) likely that the following hit is behind as well. In this respect, drumming patterns and other intentional rhythmic tasks show exactly the opposite behavior.

### Short-scale patterns

**Fig 6:**
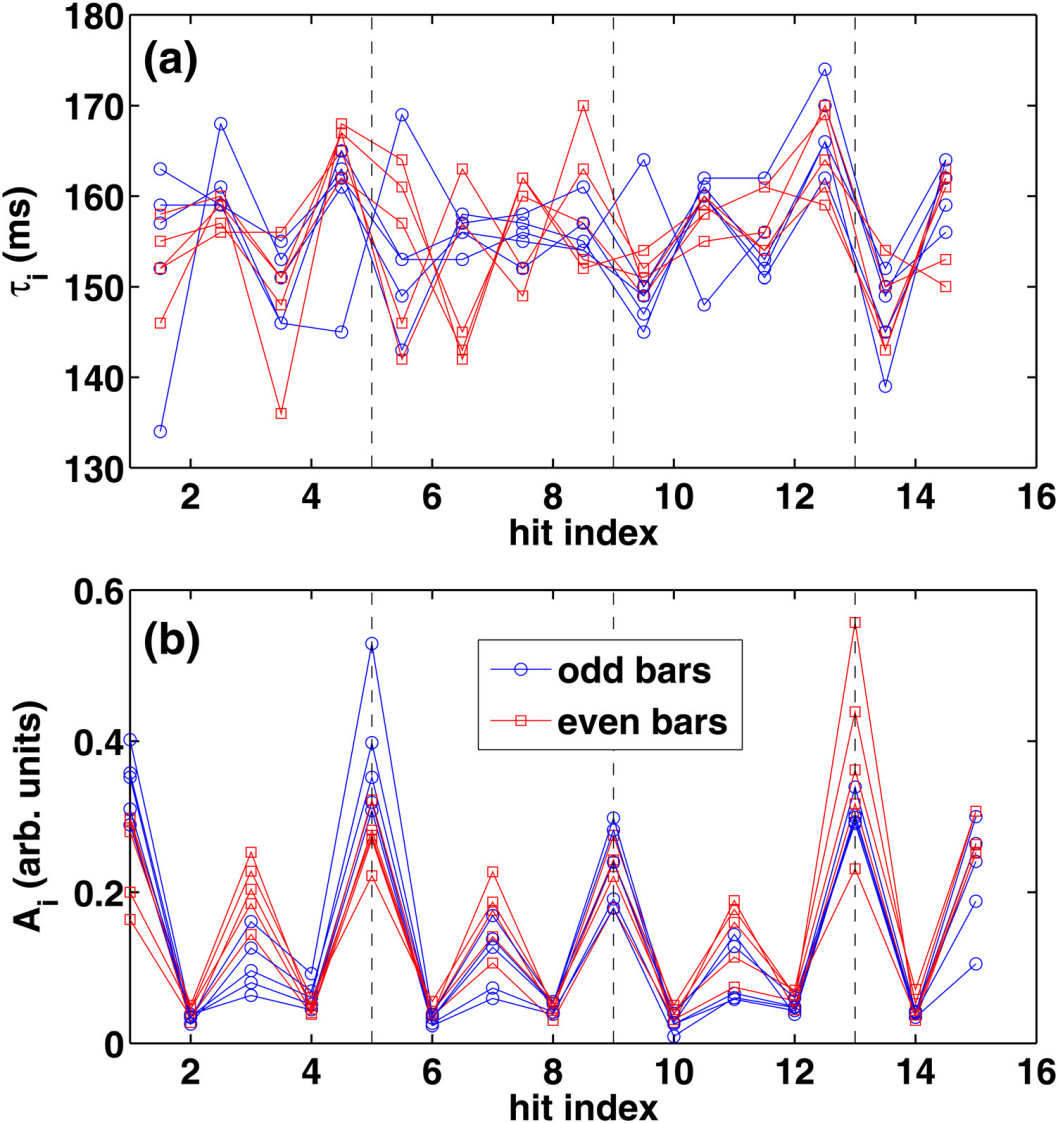
(a) 16th note interbeat intervals during the first 10 bars in “I Keep Forgettin”’. Each bar consists of 16 hits, and the corresponding intervals are marked at half-integer values. The interval between the 15th and 16th note is missing due to open hi-hats at the end of almost all bars (see text). The circles and squares correspond to odd (1, 3, …) and even (2, 4, …) bars, respectively. (b) Same as (a) but for the hit amplitudes. Again, the 16th hit is missing due to the open hi-hat—see the drum score in Fig 7.

**Fig 7:**
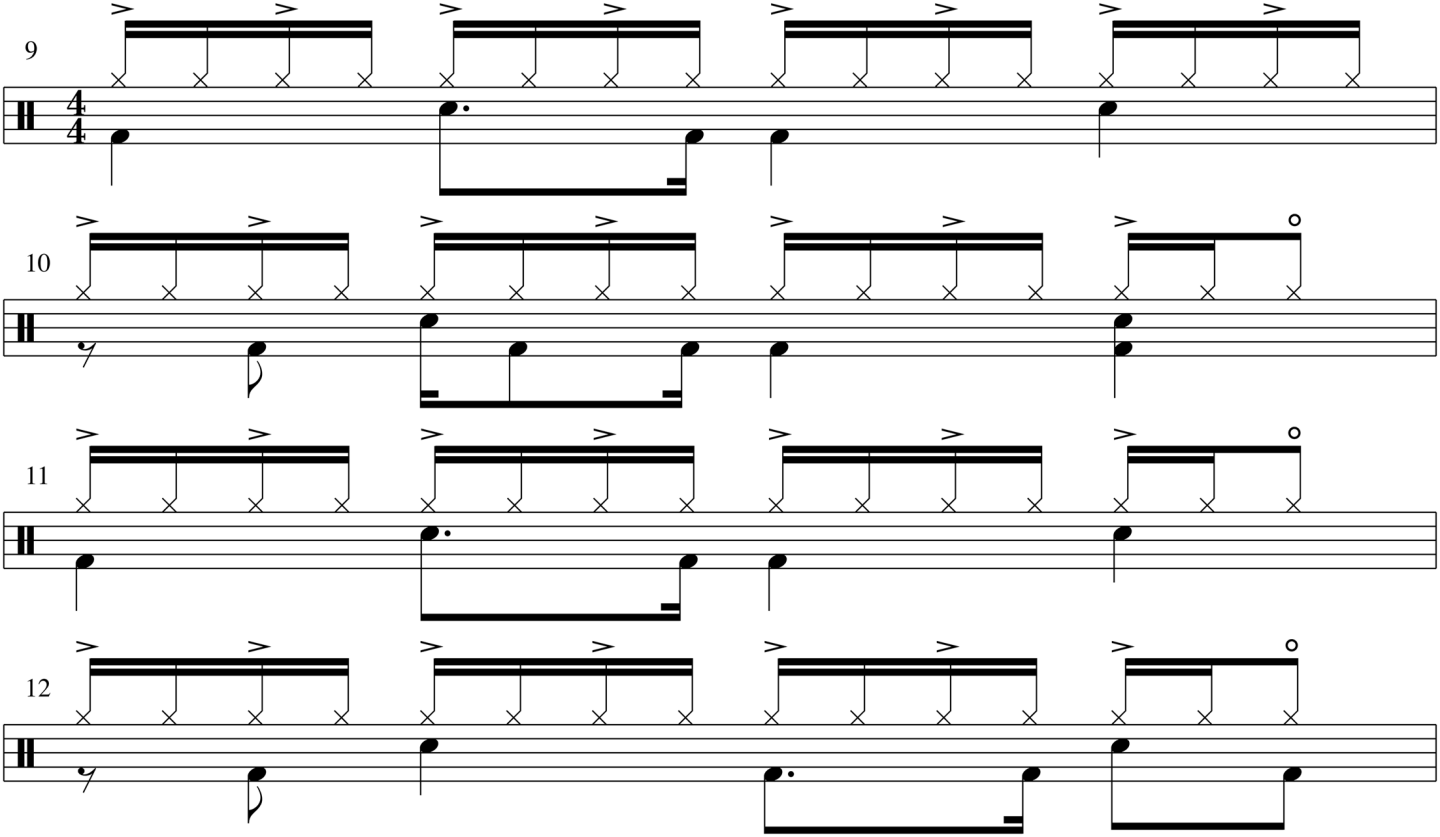
Drum score of bars 9-12 in “I Keep Forgettin”’. The 16th notes are marked on the upmost line. The (clearest) accents are marked by the symbol “>”, and the open hi-hat is marked by a symbol “o” on top of the 8th notes. The score can be compared to the detected 16th note intervals and amplitudes in Fig 6. The drum score of bars 1-4 is identical apart from the replacement of the snare by a cross-stick rimshot.

Finally we examine the short-scale interbeat and amplitude variations in the hi-hat pattern. In Fig 6 we show the hi-hat interbeat intervals (a) and amplitudes (b) of the first ten bars of the song, each consisting of 16 hits. Thus, we consider here the intro part of the song, where we have a minimal number of missing onsets. We point out that in Fig 6a the intervals are shown at *half* hit indices on the x-axis to indicate their temporal location *between* the hits. It should be also noted that hits at the last 16th note are missing, since apart from the 1st and 9th bar, there is always an open hi-hat played at the 15th note. This is shown in the drum score plotted in Fig 7 for bars 9–12. We point out that the drum score of bars 1-4 is identical apart from the replacement of the snare by a cross-stick rimshot.

In Fig 6 we have marked odd (first, third, …) and even (second, fourth, …) bars by circular and square points, respectively. As discussed above, each musical phrase of the song, characterized by the bassline, consists of *two* consecutive bars (odd+even). In Fig 6a we are able to detect clear correlations between the bars for interbeat intervals, although this effect is much more pronounced in the case of amplitudes in (b). The mean correlation coefficients $R(i,j)=C(i,j)/\sqrt{C(i,i)C(j,j)}$ (with *C*(*i*,*j*) as the covariance matrix) for all the ten bars are as high as 0.48 and 0.88 for intervals and amplitudes, respectively. Interestingly, for *only* odd (even) bars the mean coefficients are higher; 0.51 (0.53) in the case of interbeat intervals, and 0.93 (0.92) in the case of amplitudes. This difference from the mean of all the bars, albeit not remarkably large, is understandable in terms of the two-bar phrase of the song. Thus, in several ways, patterns within the phrase are subject to subtle intentional and instinctive musical interpretations. In small scales, however, the interval fluctuations may also be affected by motor delays [1] discussed above that lead to vanishing LRCs [Fig 4a and 4b].

From a musical point of view, especially the amplitudes in Fig 6b have an intriguing pattern beyond the clearly audible 8th note accenting (on the every second 16th note) which is marked in the drum score in Fig 7. The complex amplitude sequence can be described as *high—low—medium—low—very high—low—medium—low—high—low—medium—low—very high*, etc., where “very high” corresponds to the simultaneous hit on the snare with the left hand as seen in the drum score in Fig 7. The highest peaks in the last two bars in Fig 6b might be affected by a leakage from the snare signal to some extent. Nevertheless, the rest of the pattern is unaffected.

## Discussion

In summary, we have described a route to examine hi-hat patterns in real world data. In particular, we have analyzed the 16th note hi-hat intervals and amplitudes played by Jeff Porcaro in *I Keep Forgettin’*, which he plays in his unique one-handed manner. We have first generated the time-series using sensitive onset detection to one millisecond precision in the complete sound file. Then we have analyzed the drift of the sixteenth note pulse, long-range correlations (LRCs) with detrended fluctuation analysis (DFA) and spectral analysis, Poincaré maps, and finally variations on the level of one and two bars (phrase) of the song.

Our results show that the drum track was most likely recorded without the metronome, and the slight changes in the 16th note pulse reflect different parts of the song. Clear evidence of LRC fluctuations in 16th note hi-hat intervals was found. To the best of our knowledge, this phenomenon has not been found in recorded drumming in popular music before, when no metronome was present during the recording, and when no individual drum tracks were available. The LRCs seem to wash away in short time scales, likely due to motor delays studied before in human cognition. The observed anticorrelations on a small time scale, including lag-1 anticorrelations clearly visible in Poincaré return maps, are consistent with previous studies [1, 6, 9, 15]. The amplitudes also show LRC fluctuations, albeit weaker than in the case of hi-hat intervals. Our analysis of individual bars reveals complex patterns in both interbeat intervals and amplitudes. In particular, the two-bar phrase of the song is characterized by a rich amplitude pattern that goes beyond the 8th note accenting (on the every second 16th note).

Our study can be taken as the first step to analyze the complex dynamics of music recordings of the 20th and 21st century on different time scales. Several important questions arise, and in the following we mention only a few of them. First and foremost, a detailed study on LRCs in more songs, preferably of a large ensemble, would provide new insights on the nature of human timing and its relation to groove and perception of time. This may complement previous milestone studies in cognitive sciences and musicology. At present, we are constrained by the fact that the onset detection from an analog signal is a tedious task. On the other hand, available MIDI recordings that we examined so far display machine-generated (or manipulated) drum tracks *without* any interval fluctuations, which has been demonstrated to worsen the listening experience [6]. Secondly, to learn more about the groove of iconic musicians such as Jeff Porcaro, and about the universality of the considered phenomena, it would be important to compare (i) different recordings of the same drummer, (ii) different drummers, (ii) various tempos or/and rhythms, (iii) different musical genres, and (iv) playing styles, e.g., single-handed 16th note hi-hats versus the more common two-handed patterns. The last point is already under our examination. Furthermore, regarding the musical groove a major factor is the communication between players, e.g., between the drummer and the bass player [15].

In a more general context, a key question that still needs to be addressed is the *origin* of the LRCs, which are common in a variety of systems. The underlying system must be sufficiently complex, described by a nonlinear differential equation (or many of them), and there must be a proper amount of feedback. However, the origins might largely variate from system to system, and it is difficult to generate universal models that could qualitatively describe, e.g., heart-beat intervals, magnetoconductance oscillations, and drumming intervals in the same footing.

Finally we comment on possible practical applications of the present study. First, the complex (but repetitive) hi-hat patterns found here [see, e.g., Fig 6] could be implemented into drum machines in a straightforward manner to improve their “human touch”. This should be combined with LRCs that already have been subject to such proposals. [6]. Secondly, although we currently miss a comparison to two-handed hi-hat patterns, according to our results it is likely that the “single-handed method” (in Porcaro’s words [27]) is superior to the two-handed playing in order to enrich the rhythm and the feel, depending naturally on the drummer and his/her qualities. This fact should be underlined in modern pop and rock drumming pedagogy.

## Supporting Information

S1 Dataset Detected 16th note hi-hat onsets. The file contains all the detected onsets in “I Keep Forgettin”’. The onset times (in seconds) and the corresponding amplitudes (in arbitrary units) are given in the first and second column, respectively.
(TXT)
